# Small Incision Lenticule Extraction for Correction of Myopia and Myopic Astigmatism: First 24-Hour Outcomes

**DOI:** 10.1155/2017/5824534

**Published:** 2017-06-07

**Authors:** Taixiang Liu, Tingting Dan, Yan Luo

**Affiliations:** Guizhou Ophthalmic Hospital, The Affiliated Hospital of Zunyi Medical College, Zunyi 563003, China

## Abstract

**Purpose:**

To observe the first 24-hour (h) outcomes of the small incision lenticule extraction procedure (SMILE) for myopia and myopic astigmatism.

**Methods:**

Fifty-three eyes (27 patients) scheduled for SMILE were followed immediately (0 h), 2, 4, 6, and 24 h after SMILE. Uncorrected visual acuity (UCVA), conjunctival congestion, pain level, and corneal edema, thickness, and densitometry were recorded.

**Results:**

At 2 h after SMILE, 15.1% of eyes had ≤0.1 LogMAR UCVA; this increased to 62.3%, 98.1%, and 100% at 4, 6, and 24 h, respectively. Some eyes (33.96%) had mild corneal edema immediately after surgery. No 6 h postoperative edema was observed. In the first 24 h after SMILE, corneal thickness gradually decreased. Postoperative corneal densitometry values were significantly higher than preoperative values but gradually decreased during the first postoperative 24 h.

**Conclusions:**

In the first postoperative 24 h, UCVA and corneal status (edema and densitometry) improved quickly.

## 1. Introduction

Small incision lenticule extraction (SMILE) is a very important technology for correcting refractive errors in recent years [1]. Due to a smaller intraocular pressure increase during suctioning and minimal corneal and corneal nerve fiber damage, patient discomfort, the rate of dry eye, and flap-related complications could be minimized in addition to yielding a higher theoretical biomechanical advantage [2–5]. It is now becoming the most popular choice for the treatment of myopia and myopic astigmatism.

Emerging evidence has demonstrated that SMILE has good efficacy, safety, predictability, and satisfaction for patients. However, all of these data were focused on relatively long-term outcomes (often from three months to five years postsurgery) [1, 6–12]. It is well-known that visual recovery of corneal refractive surgery occurs very rapidly. Previous data has shown that SMILE recovery was slightly slower than that of laser-assisted in-site keratomileusis (LASIK), but this conclusion was derived from observations made after one or more postoperative days [13]. Some patients could even return to their jobs or have a physical examination on the same day of the surgery. Patients often paid attention to the outcomes, especially visual rehabilitation, in the first 24 h after surgery. We know that the corneal wound healing process is far from complete after 24 h, and the treating surgeon will explain to the patient that their visual acuity will still remain suboptimal. However, to our knowledge, there are still no reports about the regularity of visual rehabilitation in the first 24 h after SMILE. In consideration of the popularity of SMILE, it is necessary to observe the first 24 h postoperative outcomes. So, we designed a prospective study to observe the outcomes of SMILE immediately (0 h), 2, 4, 6, and 24 h after surgery.

## 2. Material and Methods

### 2.1. Patients and Examinations

Twenty-seven patients were consecutively enrolled in our prospective nonrandomized SMILE study. The procedures were performed at the Affiliated Hospital of Zunyi Medical College (Zunyi, China) from August to September 2016. Uncorrected visual acuity (UCVA), conjunctival congestion, pain, and corneal edema, density, and thickness 0, 2, 4, 6, and 24 h after surgery were evaluated. UCVA was recorded using the decimal method and converted into the logarithm of the minimal angle of resolution (LogMAR) equivalence.

Inclusion criteria included several parameters: (1) a minimum age of 18 years; (2) stable myopia for two years; (3) sphere ranging from −0.75 ~ −10.0 diopters (D); (4) astigmatism ranging from 0.00 to −5.00 D; (5) no corneal diseases; and (6) no history of eye diseases affecting visual function such as ocular trauma, glaucoma, cataracts, retinal diseases, and/or uveitis. The thickness of the residual stromal bed was >250 *μ*m, and the total thickness was >450 *μ*m.

This study adhered to the Helsinki declaration. After a comprehensive explanation of the possible risks of SMILE, the content, and time points of the follow-up examinations, patient informed consent was obtained as approved by the Guizhou Ophthalmic Hospital, the Affiliated Hospital of Zunyi Medical College. All participants in the study were informed of their right to withdraw from the study at anytime without any explanation. This study was a quality-control study, and all the follow-ups were done as routine examinations after any type of corneal refractive surgery, which was not a requirement for ethical approval.

Conjunctival congestion was observed and evaluated: (1) none (no signs); mild (limited congestion and bright red vessels); (3) moderate (obvious congestion, heavy red vessels with unclear borders); (4) significant (diffuse congestion and fuchsia-colored vessels without edema); or (5) severe (diffuse congestion and edema).

We evaluated the subjective symptoms of eye pains by the use of a questionnaire: (1) none (no symptoms); (2) mild (mild pain/foreign body sensation); (3) moderate (moderate pains that do not affect daily life); (4) significant (obvious pains that affect daily life but do not require painkillers); or (5) severe (severe pain that requires painkillers) [4].

The average corneal densitometry value (as detected by corneal scattering) of a 4 mm annular zone of the center of the whole cornea was collected using a Pentacam Scheimpflug camera (Oculus Optikgeräte GmbH, Wetzlar, Germany) [14]. A series of 25 images (1003 × 520 pixels) was collected. Densitometry is expressed in gray-scale units (GSUs). Zero means no corneal clouding, while a completely opaque cornea is expressed as 100. Meanwhile, the central corneal thickness was recorded using the Pentacam camera, and corneal edema was observed using slit lamp microscopy.

### 2.2. Surgical Procedure

In the present study, all surgery was performed with a VisuMax 500 kHz femtosecond laser (Carl Zeiss Meditec, Jena, Germany) by Taixiang Liu. The underside of the lenticule was cut and then followed by the lenticule sidecuts. Next, the upperside interface of the lenticule was created, and finally a 2 mm incision was created super. The lenticule was dissected using a flap separator and extracted manually via the small incision. Our SMILE parameters consisted of several parameters: (1) pulse energy 140 to 165 nJ; (2) lenticule side-cut angle 90 degrees; (3) lenticule diameter 6.5 mm; (4) cap thickness 120 mm; (5) 90-degree side-cut; and (6) circumferential length of 2.0 mm. For most cases, the procedure takes approximately 25 sec. We did not wash the intrastromal space with any solution and only washed the 2.0 mm side-cut using BBS. After the surgery, none of the patients needed eye shields. A topical antibiotic (0.3% levofloxacin, Cravit, Santen, Osaka, Japan) was administered four times per day for two weeks. A topical steroid (0.1% betamethasone, Rinderon, Shionogi, Osaka, Japan) was used four times per day for four weeks with a gradual dose reduction every other week.

### 2.3. Statistical Analysis

Numerical data are presented as mean ± SD. The normality of all data was first analyzed using the Kolmogorov–Smirnov test. The two eyes of each patient were analyzed as one cluster eye using clustered model. A repeated measure analysis of variance (ANOVA) was used to analyze the differences in vision, corneal densitometry, and central corneal thickness at different time points during the first 24 h after surgery. Visual acuity, corneal thickness, and corneal densitometry were correlated with the Pearson bivariate regression. Analyses were performed using the SPSS 17.0 software. *P* < 0.05 was considered statistically significant.

## 3. Results

All enrolled patients completed the follow-up examinations after their SMILE procedures. The preoperative characteristics of participants are described in Table 1. During the SMILE surgery, no one lost suction or had lenticule remnants. No patient had a small tear at the cap. There were no postoperative complications such as mechanical damage of corneal epithelium, infection, or diffuse lamellar keratitis.

### 3.1. Conjunctival Congestion

**Figure 1:**
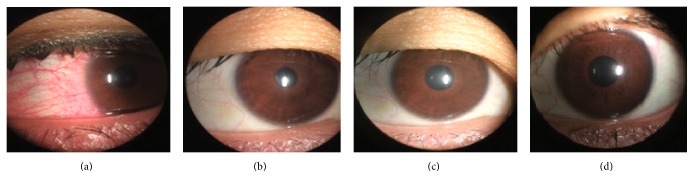
Conjunctival congestion. In our present study, it was a typical change indicating congestion immediately (a), 2 h (b), 4 h (c), and 6 h (d) after SMILE.

Almost all of the eyes had varying degrees of conjunctival congestion immediately after SMILE, but the congestion rapidly attenuated over time (Figure 1()). In our present study, none of the eyes had obvious congestion 2 h after surgery.

### 3.2. Pain/Foreign Body Sensation

We evaluated subjective symptoms of eye pains using a questionnaire (Table 2). No patient had any moderate or more severe eye pain. Even immediately after SMILE, only 20.75% eyes had mild pain or foreign body sensation. No patient had pain or foreign body sensation 6 h after surgery.

### 3.3. Visual Rehabilitation and Residual Refractive Error

UCVA (LogMAR) values were 1.27 ± 0.01, 0.39 ± 0.02, 0.25 ± 0.01, 0.14 ± 0.02, 0.03 ± 0.01, and −0.04 ± 0.01 preoperatively and 0, 2, 4, 6, and 24 h after surgery, respectively. Eyes with emmetropia as target refraction and the visual acuity quickly improved with time in the first 24 h after surgery (all *P* < 0.01). UCVA ≤0.1 LogMAR at 2 h postoperatively was seen in 15.1% of patients; this increased to 62.3%, 98.1%, and 100% at 4, 6, and 24 h, respectively (Table 3). There were no correlations between visual acuity and preoperative refractive errors (*r* = 0.150, *P* = 0.788).

The residual refractive errors were −0.24 ± 0.69 D (−1.25 ~ 0.75 D), −0.03 ± 0.58 D (−1.00 ~ 0.75 D), 0.03 ± 0.47 D (−1.25 ~ 0.75 D), 0.21 ± 0.41 D (−1.00 ~ 0.75 D), and 0.16 ± 0.30 D (−0.5 ~ 0.75 D) at 0, 2, 4, 6, and 24 h, respectively, after surgery. There were no obvious deviations in the target values around the residual refractive error. Due to all patients just accepted the operation and parts of patients had the edema cornea, we did not examine the best corrected visual acuity.

### 3.4. Corneal Edema and Corneal Thickness

Mild corneal edema existed in 33.96% eyes immediately after SMILE (Table 4). After 6 h postoperatively, there was almost no edema observed by slit lamp microscopy. Postoperatively, the central minimum corneal thickness gradually reduced over time (Table 5). Analysis using pairwise comparisons of repeated measures ANOVA revealed that there was significant differences in corneal thickness over time (all *P* < 0.01).

### 3.5. Corneal Densitometry

Table 5 shows the average corneal densitometry within the central 4 mm annular zone of the whole cornea. Compared with the preoperative corneal densitometry values, the postoperative values significantly increased and then gradually decreased over time in the first 24 h after surgery. All of the corneal densitometry peak values were located in the wounded area. Analysis using ANOVA revealed that there were significant differences in corneal densitometry at all time points (all *P* < 0.01).

Using the Pearson bivariate regression, we then analyzed the correlation between corneal densitometry, corneal thickness, and UCVA at different time points (Table 6). We found that there had no any correlation between corneal densitometry, corneal thickness, and visual acuity. No correlation was also observed between corneal densitometry and corneal thickness.

## 4. Discussion

In the present study, data showed that in the first 24 h after surgery, visual acuity improved very quickly. Even at 6 h postoperatively, most of the patients (98.1% eyes) had ≤0.1 LogMAR values.

Conjunctival congestion is the immediate postoperative symptom. In our study, there were no suction-induced conjunctival hemorrhages. Although all of the eyes had varying degrees of conjunctival congestion immediately after SMILE, it quickly attenuated over time. After 2 h postoperatively, almost all eyes had no obvious congestion. Pain often is one of worries facing any surgical patient. In fact, there was no moderate or severe eye pain after surgery. Even immediately after SMILE, only 20.75% of the eyes had mild pain or foreign body sensation, and it passed quickly over time. After 6 h postoperatively, no patients had any symptoms. It is possible that the slight symptoms after SMILE were correlated with lower vacuum suction, less tissue disturbance, and less inflammatory cell infiltration and keratocyte apoptosis in the cornea after SMILE [2, 15–18].

With SMILE being used more extensively, more patients could pay attention to the first 24 h outcome after surgery. In the present study, we found that at 2 h postoperatively, 15.1% had a UCVA of ≤0.1 LogMAR; this increased to 62.3%, 98.1%, and 100% at 4, 6, and 24 h, respectively. This indicated that in the first 24 h after SMILE, UCVA recovered quickly even though it remained suboptimal at that point. However, not comparing the early SMILE outcome with those from other corneal refractive surgery results such as photorefractive keratectomy (PRK) or LASIK was an oversight. We detected the refractive status using computer optometry and found the residual refractive errors were −0.24 ± 0.69 D (−1.25 ~ 0.75 D), −0.03 ± 0.58 D (−1.00 ~ 0.75 D), 0.03 ± 0.47 D (−1.25 ~ 0.75 D), 0.21 ± 0.41 D (−1.00 ~ 0.75 D), and 0.16 ± 0.30 D (−0.5 ~ 0.75 D) at 0, 2, 4, 6, and 24 h after surgery, respectively, with no obvious deviations in the target value. In addition, the correlation between UCVA and preoperative refraction was analyzed and no correlation was found. We speculated that both the preoperative refraction and residual refraction were not major factors impacting visual rehabilitation in the first 24 h after SMILE.

Temporary corneal edema is one of the common signs during the early stages after corneal refractive surgery. Using slit lamp microscopy, we observed that SMILE induced mild corneal edema in 33.96% of the eyes immediately after surgery, but none of the eyes had moderate or more corneal edema. Given that sometimes slight corneal edema could not be discerned using slit lamp microscopy and central corneal thickness can be used to quantitatively evaluate the corneal edema, we also investigated the central minimum corneal thickness using the Pentacam camera [19]. Data showed that in the first 24 h after SMILE, the central minimum corneal thickness gradually decreased over time, and the differences in corneal thickness were significant between any postoperative pair-wise comparisons. Corneal densitometry is an objective quantitative assessment that is performed by examining corneal light scatter, which was an important method for evaluating the corneal status in some eye diseases such as corneal transplant, corneal cross-linking, and corneal refractive surgery [20–22]. This parameter is regarded as an indicator of corneal transparency [23]. During the early stages after corneal refractive surgery, corneal densitometry increased, then gradually declined, and had returned to preoperative values by 12 months after surgery [22, 24]. In normal corneas, densitometry values are derived from the total cornea with the corneal epithelium as the greatest contributor [23]. In this study, a baseline value (preoperative densitometry) of 13.31 ± 0.99 GSU was in agreement with findings from recent studies [25, 26]. In the first 24 h, SMILE significantly increased corneal densitometry and all sites of densitometry peak values located in the wounded area. It was difficult to clearly explain the increased corneal densitometry. There were several factors that had an impact on corneal densitometry in the first 24 h after SMILE. (1) Bubble: from immediately to 4 h after SMILE, corneal clarity rapidly improved, which may have been related to quick reabsorbtion of the femtosecond laser-induced bubble. Subsequently, the recovery of corneal densitometry values reached a relatively slow phase and then returned gradually back to the baseline values. (2) Edema: Previous study showed that edema cornea could increase the light scatter [27]. However in our study, no any correlation was found between corneal densitometry and corneal thickness, which was similar to Garzón and colleagues' study [14]. Maybe the thickness could not completely reflect the level of corneal edema. (3) Particle: no particles were observed by slit microscopy in our study after SMILE. Using transmission electron microscopy, Wei et al. found that cell debris was visible in the damaged area using transmission electron microscope 1 day postoperatively after SMILE [28]. This debris could act as a tiny mirror causing random reflection followed by an increase in light scattering [24, 29]. (4) Inflammation: although in our study in the first 24 h after SMILE, no patient had any sign of inflammation, especially diffuse lamellar keratitis. Previous studies have suggested that femtosecond laser alone could induce corneal inflammation and haze, which could increase corneal densitometry with a peak in keratocyte apoptosis at approximately 4 h after injury [30, 31]. Thus, we could not rule out its impact on the corneal densitometry measurements in the first stages after SMILE [24].

After corneal refractive surgery, postoperative corneal status may be one of the factors impacting visual rehabilitation. So we analyzed the correlation between corneal thickness, corneal densitometry, and UCVA. Interestingly, although in the first 24 h after SMILE, corneal densitometry and thickness both significantly increased, there was no correlation between visual acuity and corneal densitometry or corneal thickness at any time point, which could be due to the small number of patients.

In conclusion, visual acuity remained suboptimal in the 24 h after SMILE, but it improved quickly. Even at 6 h postoperatively, most of patients (98.1% eyes) had a UCVA of ≤0.1 LogMAR for UCVA. The corneal status, especially corneal densitometry and corneal thickness, also could be improved quickly.

## Figures and Tables

**Table 1:** Preoperative baseline characteristics of participants.

Sex	Male (12)	Female (15)
Age (y)	24.60 ± 5.65 years (range, 18–37 years)	
Sphere	−4.91 ± 1.20 D (range, −2.75 D to −7.50 D)	
Cylinder	−0.86 ± 0.50 D (range, 0 D to −2.25 D)	
Spherical equivalent refraction	−5.19 ± 1.17 D) (range, −3.0 D to −7.63 D)	

**Table 2:** Questionnaire regarding the subjective symptoms of eye pain.

Pain/foreign body sensation	Immediate	2 h	4 h	6 h	24 h
None	42	43	49	53	53
Mild	11	10	4	0	0
Moderate	0	0	0	0	0
Significant	0	0	0	0	0
Severe	0	0	0	0	0

**Table 3:** Visual acuity after SMILE (53 eyes of 27 patients).

Decimals	5'	Snellen (20 ft)	LogMAR	0		2 h		4 h		6 h		24 h	
				*n* %									
0.1	4.0	20/200	1.0										
0.12	4.1	20/160	0.9										
0.15	4.2	20/125	0.8										
0.2	4.3	20/100	0.7										
0.25	4.4	20/80	0.6	10	18.9								
0.3	4.5	20/63	0.5	7	13.2	2	3.8						
0.4	4.6	20/50	0.4	9	17.0	4	7.6	1	1.9				
0.5	4.7	20/40	0.3	18	34.0	12	22.6	7	13.2	1	1.9		
0.6	4.8	20/32	0.2	7	13.2	27	50.9	12	22.6				
0.8	4.9	20/25	0.1	2	3.8	81	5.1	26	49.1	17	32.1	2	3.8
1.0	5.0	20/20	0.0					6	11.3	26	49.1	22	41.5
1.2	5.1	20/16	−0.1					1	1.9	9	17.0	29	54.7
1.5	5.2	20/13	−0.2										
2.0	5.3	20/10	−0.3										

**Table 4:** Corneal edema after SMILE (53 eyes of 27 patients).

Edema	0	2 h	4 h	6 h	24 h
None	35	40	50	53	53
Mild	18	13	3	0	0
Moderate	0	0	0	0	0
Significant	0	0	0	0	0
Severe	0	0	0	0	0

**Table 5:** Corneal thickness and densitometry pre- and postoperatively.

Time point	Corneal thickness (*μ*m)	Corneal densitometry (GSU)
Preoperative	541 ± 26	13.31 ± 0.99
0	494 ± 34	19.22 ± 2.40
2 h	481 ± 37	18.25 ± 3.46
4 h	467 ± 34	16.66 ± 2.36
6 h	461 ± 32	15.81 ± 1.95
24 h	450 ± 27	14.88 ± 1.41

**Table 6:** Correlation between corneal densitometry, corneal thickness, and visual acuity.

Time point	UCVA and densitometry		UCVA and thickness		Densitometry and thickness	
	Pearson correlation	*P* value	Pearson correlation	*P* value	Pearson correlation	*P* value
Preoperative	−0.006	0.976	0.207	0.301	−0.008	0.970
Immediate	−0.168	0.403	−0.023	0.911	−0.087	0.667
2 h	0.468	0.053	−0.023	0.910	0.277	0.161
4 h	0.372	0.056	−0.080	0.691	0.114	0.573
6 h	0.341	0.081	0.166	0.408	0.007	0.974
24 h	0.274	0.167	−0.211	0.291	−0.270	0.174
